# The NF-κB Signaling Pathway, the Microbiota, and Gastrointestinal Tumorigenesis: Recent Advances

**DOI:** 10.3389/fimmu.2020.01387

**Published:** 2020-06-30

**Authors:** Chao Peng, Yaobin Ouyang, Nonghua Lu, Nianshuang Li

**Affiliations:** ^1^Department of Gastroenterology, The First Affiliated Hospital of Nanchang University, Nanchang, China; ^2^Institute of Digestive Disease, The First Affiliated Hospital of Nanchang University, Nanchang, China

**Keywords:** NF-κB signaling pathway, *Helicobacter pylori*, microbiota, gastric cancer, colorectal cancer

## Abstract

Gastrointestinal (GI) cancers, especially gastric cancer and colorectal cancer (CRC), represent a major global health burden. A large population of microorganisms residing in the GI tract regulate physiological processes, such as the immune response, metabolic balance, and homeostasis. Accumulating evidence has revealed the alteration of microbial communities in GI tumorigenesis. Experimental studies in cell lines and animal models showed the functional roles and molecular mechanisms of several bacteria in GI cancers, including *Helicobacter pylori* in gastric cancer as well as *Fusobacterium nucleatum, Escherichia coli, Peptostreptococcus anaerobius*, and *Bacteroides fragilis* in CRC. The transcriptional factor NF-κB plays a crucial role in the host response to microbial infection through orchestrating innate and adaptive immune functions. Moreover, NF-κB activity is linked to GI cancer initiation and development through its induction of chronic inflammation, cellular transformation and proliferation. Here, we provide an overview and discussion of modulation of the NF-κB signaling pathway by microbiota, especially infectious bacteria, in GI tumorigenesis, with a major focus on gastric cancer and CRC.

## Introduction

Cancer is the second leading cause of death globally behind cardiovascular disease, according to statistical data from the World Health Organization (1). Gastrointestinal carcinoma remains the main cause of cancer-related morbidity and mortality worldwide, particularly in East Asian countries (2). The roles of genetic risk factors in cancer development have been well-studied. Germline mutation in CDH1 (E-cadherin) is widely detected in gastric cancer (3). The genetic mutation of adenomatous polyposis coli (APC) is associated with a higher risk of familial adenomatous polyposis and colorectal cancer (4). In addition, abundant and diverse microbes reside in the human body. These microorganisms include bacteria, fungi, archaea, and viruses. Approximately 100 trillion of microorganisms exist in the human gastrointestinal tract (5, 6). The activities of complex microbial communities orchestrate many aspects of human health, such as immune responses, metabolic balance, and homeostasis. Recently, accumulating evidence suggests that disruption of the microbiota is involved in diverse human diseases, including gastrointestinal disorders, obesity, inflammatory bowel disease (IBD), and depression (7, 8). Data at different levels from animal models and cell lines indicate that microbial pathogens exert oncogenic properties during gastrointestinal tumorigenesis (9, 10). It has been well-established that infection with the gram-negative bacterium *Helicobacter pylori* (*H. pylori*) significantly increases the risk of gastric cancer. The presence of *Fusobacterium nucleatum* (*F. nucleatum*), a gram-negative obligate anaerobic bacterium, can contribute to intestinal tumorigenesis (11). The NF-κB signaling pathway can be activated to modulate host cellular events after exposure to different microbial pathogens or microbial products, such as lipopolysaccharide (LPS) and pathogen-associated molecular patterns (PAMPs) (12). Cytoplasmic NF-κB is transferred to the nucleus, where it induces antimicrobial inflammatory cytokine expression, which functions as a rapid defense mechanism against microbes, including infectious bacteria. However, prolonged chronic inflammation due to the activation of NF-κB proteins may result in tissue damage, further contributing to tumorigenesis by changing the genetic and epigenetic states of damaged tissues and the host microenvironment (13). In this review, we provide an update on recent advances in our understanding of the modulation of the NF-κB signaling pathway by microbes, particularly infectious bacteria in gastrointestinal tumorigenesis, with a major focus on stomach and intestinal cancers.

## Signal Transduction of the NF-κB Pathway

### Activation of NF-κB Signaling

**Figure 1:**
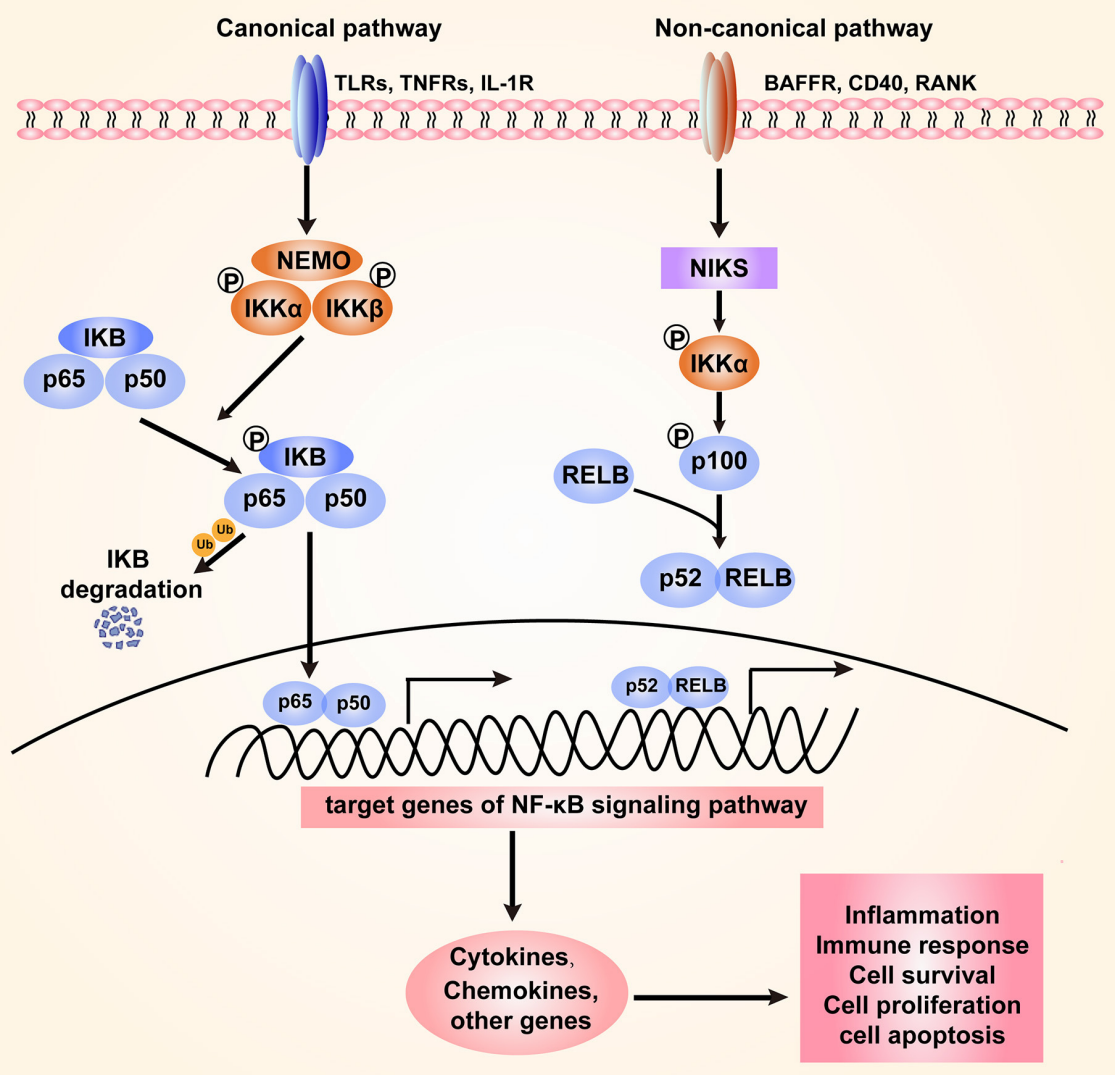
The canonical and non-canonical NF-κB signaling pathway. The canonical pathway is induced by TLRs, TNFRs, and IL-1R. Activation of this cascade leads to the phosphorylation and degradation of inhibitory protein IκB. NF-κB is activated by release from the IκB-containing complex, then translocating into nucleus. The non-canonical pathway is dependent on the activation of NF-κB2 (p100)/ RelB complex by BAFFR, CD40, and RANK. This cascade induces phosphorylation of NIK, which subsequently phosphorylates IKKα. Then p52-RelB heterodimer is activated and translocate to the nucleus. The activation of NF-κB signaling regulates various cellular processes through targeting the expression of cytokines, chemokines and other genes.

The NF-κB family of transcriptional factors regulates a large number of genes involved in different cellular processes, such as cell proliferation, differentiation, genome stability, and the innate immune and adaptive immune responses (14). The NF-κB family consists of five members that interact with each other to homodimerize or heterodimerize: NF-κB1 (also named p50), NF-κB1 (also named p52), RelA (also named p65), RelB, and c-Rel (15). Activation of the NF-κB signaling pathway can occur through canonical and non-canonical (or alternative) pathways (16). The IKK kinase complex, including the catalytic subunits IKKα, IKKβ, and a regulatory subunit NF-κB essential modulator (NEMO), is the core component of the NF-κB signaling cascade (17). Under normal physiological conditions, NF-κB dimers in an inactive form are sequestered to the cytoplasm through their interaction with IKB-inhibitory proteins (IκBα, IκBβ, and IκBε). Upon stimulation with diverse bacteria, various immune receptors, such as Toll-like receptors (TLRs) and TNF receptors (TNFRs), can be activated to mediate the NF-κB signaling pathway. The primary mechanism of canonical NF-κB activation is the degradation of IκBα. In this process, IKK phosphorylates IκBα and leads to its ubiquitination through the SCF^βTrCP^ ligase-dependent proteasome degradation machinery. As a result, NF-κB is released and translocated from the cytoplasm to the nucleus, where it binds DNA and regulates downstream gene transcription (18–20). The alternative NF-κB signaling is mainly dependent on the activation of NF-κB2 (p100)/ RelB complex, which specifically responds to a subset of receptors, including BAFF (B-cell activating factor belonging to TNF family) receptor (BAFFR), CD40, and receptor activator for NF-κB (RANK) (21). NF-κB-inducing kinase (NIK) is a core component of the non-canonical pathway. Ikkα is activated by NIK and then phosphorylates p100 (22). Then, p100 is processed to its active form, p52, which forms a heterodimer with RelB that translates to the nucleus (21, 23–25). Both the canonical and non-canonical pathways can be mediated to orchestrate host inflammation in response to microbial pathogen infection (Figure 1).

### NF-κB Activation, Inflammation and Cancer

Activation of the NF-κB cascade is a central regulator of host responses to microbial infection. The innate immune response, a first line of host defense against different microorganisms, is modulated by the NF-κB signaling pathway, which in turn promotes the expression of target genes (12, 26). Most importantly, NF-κB acts as a central regulator of the immune response and inflammation by upregulating many chemokines (CXCL1, CXCL2, CXCL3, etc.) and cytokines (TNFα, IL-1β, IL-6, IL-8, etc.) (27). Activated NF-κB also affects cellular proliferation and apoptosis by targeting Bcl2, IAPs, and cyclins. In addition, NF-κB is essential for the induction of antimicrobial effectors that can effectively eliminate pathogenic microbes, such as antimicrobial peptides (AMPs) (28).

NF-κB, a critical regulator, has been linked to inflammation and cancer at multiple levels (13, 29). On the one hand, inflammation is a host-protective response to microbial pathogens or tissue damage. Upon stimulation by diverse bacterial species (*H. pylori, F. nucleatum, etc*.), NF-κB is highly activated at the site of infection for its antimicrobial activity and maintenance of tissue homeostasis (30). On the other hand, the strong involvement of the NF-κB pathway in the adaptive immune response, through either B or T cells, increases the severity and extent of inflammation (31). Constitutive chronic inflammation may lead to damaged tissues, autoimmune diseases and cancer initiation by increased cellular stresses and the accumulation of DNA damage. The alteration of genetic stability and epigenetic states at the site of damaged tissues contributes to generating a pro-tumorigenic microenvironment (32). Elevated NF-κB activity and the increased expression of proinflammatory cytokines have been documented in various tumorous tissues (33, 34). Constitutive activation of NF-κB turns on the transcription of genes that promote cell proliferation, cell survival, and genomic instability and thereby contributes to oncogenic mutations. There is strong evidence that the inducible activation of the NF-κB cascade promotes cell proliferation by targeting cyclin D_1_ expression and inhibits cell apoptosis by targeting BCl_2_ expression (35, 36). NF-κB can be activated following DNA damage. The activation of NF-κB triggers acute and chronic inflammation, which in turn is linked to decreased genomic stability and genetic mutations in cancer initiation and progression (37). As a result, the NF-κB signaling pathway is believed to play an important role in the pathogenesis and carcinogenesis of microbial infection. Here, we focus on the bacteria that can cause gastrointestinal cancer by modulating the NF-κB signaling pathway.

## Bacterial Pathogens Linked to Gastrointestinal Tumorigenesis

### The Gastric Microbiota and Gastric Cancer

No bacterium was known to specialize in colonization of the human stomach, with its unique acid environment, until the discovery of the gram-negative bacterium *H. pylori*, which was first reported in the stomachs of patients with peptic ulcers in 1982 (38). To survive in acidic conditions, *H. pylori* produces a large amount of the enzyme urease, which catalyzes the hydrolysis of urea to ammonia, thereby neutralizing gastric acid (39). Approximately half of the world's population is infected with *H. pylori*, mainly in developing countries (40). *H. pylori* infection has been extensively studied and found to be associated with an increased risk of gastric adenocarcinoma. Long-term infection with *H. pylori* is an inducible factor leading to gastric atrophic gastritis, intestinal metaplasia, dysplasia, and ultimately gastric cancer, a sequence also called the Correa cascade of multistep gastric carcinogenesis (41). Accumulating data from clinical follow-up studies suggest that eradication of *H. pylori* significantly reduces the risk of gastric cancer (42, 43). This is illustrated by the finding that patients have a lower incidence of metachronous gastric cancer following treatment to eradicate *H. pylori* (44). Additionally, in patients with *H. pylori* infection who had a family history of gastric carcinoma in their first-degree relatives, *H. pylori* eradication significantly decreased gastric cancer risk (45).

Some heterogeneity exists between different *H. pylori* strains. High prevalence of *H. pylori* infection, but low prevalence of GC incidence, was found in many African countries (46). Multiple mechanisms are involved in the interaction between the host and pathogenic *H. pylori*. Both bacterial and host genetic factors contribute to *H. pylori* infection-induced chronic inflammation, metaplasia and gastric tumorigenesis (47). From the perspective of bacteria, the virulence factors of *H. pylori* have been demonstrated to influence this microorganism's pathogenicity. Cytotoxin-associated gene A (CagA) and vacuolating cytotoxin A (VacA), the most intensively investigated virulence factors, play significant roles in gastric adenocarcinoma induced by *H. pylori* infection. The bacterium utilizes a type IV secretion system (T4SS) to inject CagA into host gastric epithelial cells. As a result, CagA is responsible for the dysregulation of cellular proliferation and apoptosis through disturbing the PI3K/AKT, MEK/ERK, and Wnt/β-catenin signaling pathways (48). Additionally, it has been indicated that CagA induces an inflammatory response via activation of the NF-κB pathway (49). In addition, the VacA toxin of *H. pylori* can rapidly cause vacuolation in gastric epithelial cells (50). From the perspective of host genetics, gene polymorphisms can increase the risk of gastric cancer in patients with *H. pylori* infection. Polymorphisms in the IL-1β gene increase the risk of gastric carcinogenesis in *H. pylori*-positive populations (51).

With the development of sequencing platforms and bioinformatics tools, 16S rRNA gene sequencing based on hypervariable regions was performed to study the profile of the human microbiome, including mainly the microbiotas of the stomach and intestine (52). As a result, many gastric bacteria species other than *H. pylori* have been identified. The stomachs of *H. pylori*-positive and *H. pylori*-negative individuals exhibit significantly different bacterial communities. Among *H. pylori*-positive patients, *H. pylori* is the most dominant bacterium in the stomach. In contrast, the gastric microbiota of *H. pylori*-negative individuals is more diverse and consists mainly of *Firmicutes, Proteobacteria, Bacteroidetes, Fusobacteria, and Actinobacteria* (53). Recently, gastric bacterial communities were shown to be associated with gastric malignancies. Ferreira et al. showed that *Firmicutes* and *Actinobacteria* are over-represented in the gastric carcinoma microbiota compared with the chronic gastritis microbiota. Furthermore, gastric cancer samples exhibit a significant reduction in the abundance of *H. pylori* (54). Coker et al. identified differences in mucosal bacterial interactions across stages of gastric carcinogenesis, from superficial gastritis to atrophic gastritis, intestinal metaplasia, and GC. The significant enrichment and central network locations of five microbes (*Peptostreptococcus stomatis, Streptococcus anginosus, Parvimonas micra, Slackia exigua*, and *Dialister pneumosintes*) suggest their important role in GC progression (55).

The effect of non-*H. pylori* bacteria on gastric pathology is further supported by animal model systems. In transgenic INS-GAS mice with high circulating gastrin levels, colonization of *H. pylori* led to a significant increase in *Firmicutes* and reduction in *Bacteroidetes* (41). Other *Helicobacter* species, such as *Helicobacter felis* (*H. felis*), commonly colonize animals. Mongolian gerbils infected with *H. felis* developed premalignant gastric lesions (56). Moreover, germ-free transgenic INS-GAS mice supplemented with normal intestinal flora (IF) or 3 species of commensal bacteria (rASF; ASF356 *Clostridium* species, ASF361 *Lactobacillus murinus*, and ASF519 *Bacteroides* species) developed more severe gastric lesions and elevated levels of proinflammatory genes than *H. pylori*-monocolonized INS-GAS mice (57). These findings suggest that microbial diversity contributes to gastric cancer risk. Long-term *H. pylori* infection causes gastric atrophy, which leads to achlorhydria and decreased acid secretion. Notably, *H. pylori* infection and alteration of the acidity of the gastric environment may result in alterations in the gastric microbiota (58). However, due to the difficulty in bacterial isolation and culture, the functional role and pathogenic mechanisms of microbial communities in gastric neoplasia remain unclear.

There are some genetic, environmental, dietary, and lifestyle factors that influence microbiome system. Genetic mutation such as CDH1 and TP53, lifestyles including smoking, low fruits and vegetables consumption, high salts, nitrates, and pickled foods intake and overweight are also found to be associated with increased GC risk (59, 60). He et al. reported that 12 week high-fat diet lead to the dysbiosis of gastrointestinal microbiota in C57BL/6 mice, what's more, the alterations of microbiota in stomach was earlier than that in gut and the dysbiosis of gastrointestinal microbiota may related with the metabolic disorders of mice (61). Arita et al. showed that high-fat diet leads to severe dysbiosis of gastric microbiota and increased gastric leptin, which lead to the development of gastric intestinal metaplasia in C57 mice (62). A recent large scale blinded randomized placebo controlled trial in China showed that both *H. pylori* treatment and vitamin supplementation lead to a significant reduced incidence of GC, and *H. pylori* treatment, vitamin or garlic supplementation lead to a significant reduction of GC mortality (42).

### Imbalance Between Gut Microbiota and Intestinal Cancer

Changes in the gut microbiota, referred to as dysbiosis, are involved in the development of multiple diseases, including cancer, metabolic diseases, cardiovascular diseases and depression (63, 64). Colorectal cancer (CRC) is the third most common cancer worldwide (2, 65). Effects of the fecal microbiota transplant of human fecal samples to germ-free mice suggested the effect of gut microbiota on colorectal carcinogenesis. Germ-free mice gavaged with stool from patients with CRC showed increased levels of proinflammatory genes, including the chemokines CXCR1 and CXRC2 as well as the cytokines IL-17A, IL-22, and IL-23A (66). Sequencing studies have revealed discrepancies in the gut microbiomes of patients with colorectal cancer and healthy individuals. 16S rRNA sequencing data from stool samples from CRC patients suggested that several genera, including *Fusobacterium, Porphyromonas, Enterococcus, Escherichia, Klebsiella, Streptococcus*, and *Parvimonas*, are linked to CRC (67–69). Hibberd et al. analyzed the intestinal microbiota from tumor tissues and the normal mucosa. Several taxa, including *Fusobacterium*, Selenomonas and Peptostreptococcus, were selectively enriched in patients with colon cancer compared to control individuals. Probiotic intervention significantly altered the microbial composition (70). Furthermore, significant differences in microbial communities were observed across the stages of colorectal carcinogenesis. Nakatsu et al. compared the mucosal microbiotas of normal tissues, adenomatous polyps and carcinomas. *Fusobacterium, Parvimonas, Gemella*, and *Leptotrichia* were significantly increased in patients with adenoma, which is an early-stage CRC, whereas the abundance of bacterial communities, including *Bacteroides, Blautia*, and *F. prausnitzii*, was decreased. In late-stage CRC, neither of these changes was significant. Furthermore, *Peptostreptococcus* and *Parvimonas* showed a strongly positive relationship in colonic carcinoma and carcinoma-adjacent mucosa (71). In addition, Warren et al. compared the CRC tissues and matched normal control tissues. Co-occurrence network analysis was performed to identify microbe-microbe and host-microbe associations specific to tumors. The authors confirmed tumor over-representation of *Fusobacterium species* and observed significant co-occurrence within individual tumors of *Fusobacterium, Leptotrichia*, and *Campylobacter species* (72). As a corollary, these studies may identify novel CRC-associated microbial markers, such as *Fusobacterium, Peptostreptococcus, Leptotrichia*, and *Parvimonas*.

Recently, an increasing number of studies have elucidated the functional roles and molecular mechanisms of several specific bacterial species in CRC carcinogenesis, including *Fusobacterium nucleatum* (*F. nucleatum*), *Escherichia coli* (*E. coli*), *Peptostreptococcus anaerobius* (*P. anaerobius*), and *Bacteroides fragilis* (*B. fragilis*) (**Figure 3**). *F. nucleatum* is highly increased in CRC patients compared to healthy individuals, and its abundance is closely related to a worse survival rate (73, 74). Biological informatic, functional and mechanistic studies in human samples, cell lines and animal models have revealed the potential role of *F. nucleatum* in CRC chemotherapy. Specifically, these bacteria could promote CRC resistance to chemotherapy through the activation of autophagy mediated by TLR4/MYD88 and miRNAs (75). These studies suggest that *F. nucleatum* serves as a candidate prognostic biomarker in CRC. Although *E. coli* is a commensal bacterium that colonizes the human GI tract, several studies indicate a link between some pathogenic *E. coli* strains and CRC risk. *E. coli* are divided into 4 phylotypes, among which specific *E. coli* strains from each phylotype have been associated with IBD, which is a known risk factor for CRC (76, 77). Infection with pathogenic *E. coli* in multiple intestinal neoplasia (Min) mice significantly increased colonic polys compared with those in control groups (78). Pathogenic *E. coli* produces various virulence factors. Colibactin, a hybrid polyketide-peptide encoded by the *pks* genomic island, has been shown to induce DNA double-strand breaks and genomic instability in human cells (79), contributing to mutational signatures in CRC (80). In addition, the anaerobic bacterium *P. anaerobius*, which is enriched in the fecal and mucosal samples of CRC patients, was recently demonstrated to promote CRC carcinogenesis through *in vitro* and *in vivo* studies (81). The gram-negative bacterium *B. fragilis* is a normal commensal bacterial species that colonizes the colon. A subset of *B. fragilis* termed enterotoxigenic *B. fragilis* produces the *B*. *fragilis* toxin (*BFT*) and has been found to play an important role in CRC tumorigenesis and development. Sears et al. revealed the increased abundance of the *BFT* gene in mucosal biopsies from patients with CRC compared to normal individuals. Furthermore, increased *bft* positivity was found in early to progressive CRC patients (82). This research team then investigated the role of the gut microbiota in the development of familial adenomatous polyposis (FAP), which is an autosomal dominant disease caused by the *APC* gene in which many colorectal adenomas can develop (83). The genes colibactin from *E. coli* and *bft* from *B. fragilis* are more highly expressed in patients with FAP compared to healthy individuals. Mechanistically, the co-colonization of *E. coli* and *B. fragilis* in the colon promotes the expression of inflammatory cytokines and accelerates DNA damage (84). As a result, modulation of the gut microbiota may be an effective strategy for the prevention or treatment of CRC.

Some genetic, diet, lifestyle and other environmental factor have been shown to modulate gut microbiota, further affect host metabolism, immune response and promote colorectal carcinogenesis. Liang et al. reported that APC mutation was closely related to the alteration of gut microbiota that plays an important role in the development of CRC from intestinal adenomatous polyps (85). The increasing incidence of CRC in western countries is partly attributed to the increasing adoption of western lifestyles and overweight. High fat diet is a well-known factor to influence the gut microbiota and promote CRC development in animal model (86). Interestingly, it has been demonstrated that Mediterranean Diet (MD) could counteract CRC that caused by high-fat diet by modulate apoptosis and gut microbiota in mice (87).

## Modulation of the NF-κB Signaling Pathway in Response to Bacterial Infection in Gastric Tumorigenesis

**Figure 2:**
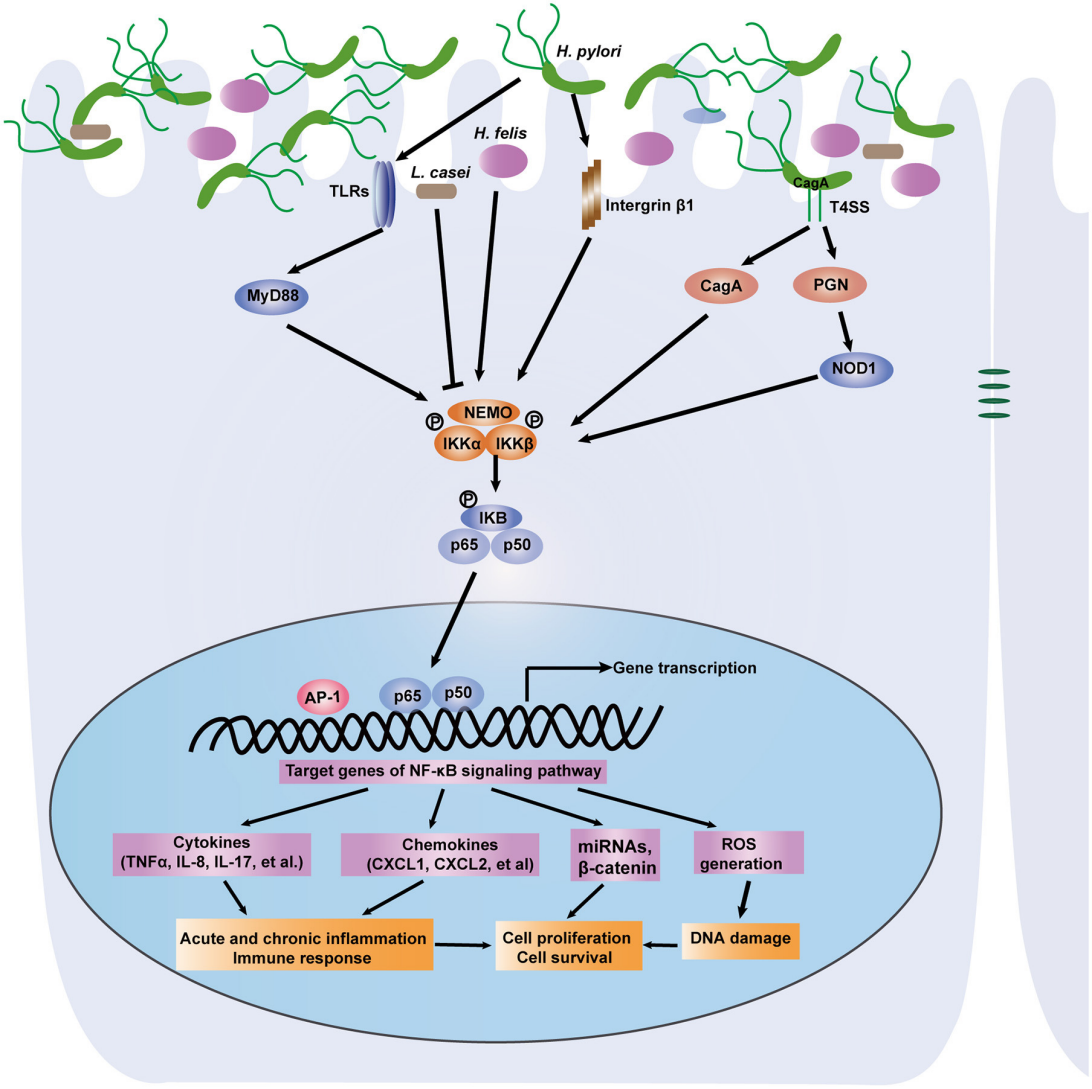
A depiction of the modulation of NF-κB signaling pathway by gastric microbiota, especially *H. pylori*. Gastric epithelial cells PRRS (NOD1, TLRs) specifically recognize the virulence factors such as CagA and peptidoglycan that are delivered by the *H. pylori* cag pathogenicity island. Then the NF-κB pathway is activated through IKK-mediated phosphorylation of IκB. Active NF-κB translocate to nucleus and induce downstream genes expression. NF-κB activation promotes acute, chronic inflammation and immune response via induction of cytokines and chemokines. The accumulation of DNA damage is associated with activation of the NF-κB signaling pathway. Several miRNAs and other genes can be activated. As a result, *H. pylori* infection induces gastric carcinogenesis via regulation of cell proliferation and survival. Other microorganisms including *L. casei* and *H. felis* link the NF-κB pathway to gastric inflammation and tumorigenesis.

**Figure 3:**
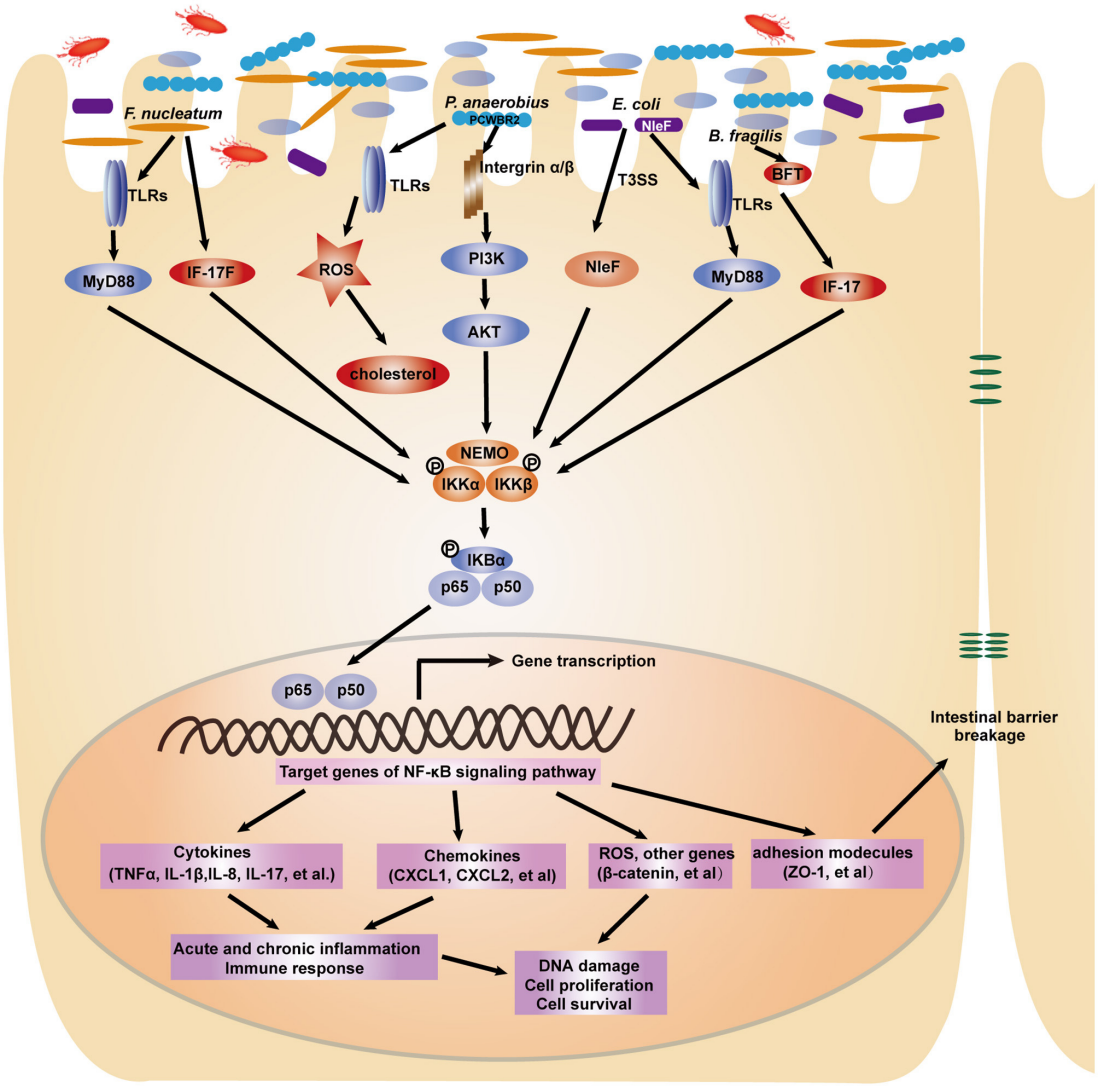
A depiction of the modulation of NF-κB signaling pathway by gut microbiota in colorectal tumorigenesis. In response to intestinal bacterial pathogens including *F. nucleatum, P. anaerobius, E. coli, B. fragilis*, intestinal epithelial cells employ several receptors such as TLRs and integrins to recognize distinct microbial components. The NF-κB signaling pathway is subsequently activated to induce the expression of pro-inflammatory cytokines, chemokines, adhesion molecules and other genes, which ultimately lead to the cellular processes changes and contribute to colorectal carcinogenesis.

Commensal microbes in the GI tract are essential for the maintenance of GI functions, including development, immune responses and homeostasis. It is becoming increasingly clear that disruption of the microbiota contributes to gastric and intestinal tumorigenesis. In particular, upon infection with intestinal bacteria, host cells rapidly employ the intracellular NF-κB signaling pathway to activate antibacterial immunity and maintain intestinal barrier integrity (30). A number of studies have reported that during bacterial infection, the NF-κB pathway controls multiple cellular processes, including inflammation, proliferation and apoptosis, by regulating the expression of a network of downstream effectors. As a result, the NF-κB signaling pathway is strongly involved in microbiota-associated gastric (Figure 2) and colorectal tumorigenesis (Figure 3). Here, we focus on the bacteria that modulate the NF-κB pathway in gastrointestinal tumorigenesis.

### *H. pylori* Infection, the NF-κB Signaling Pathway, and Gastric Carcinogenesis

*H. pylori*-infected individuals develop acute or chronic gastritis, and in a subset of subjects, gastritis may progress to peptic ulcer disease, in particular, intestinal metaplasia and gastric carcinomas (88). There is very compelling evidence for the involvement of the NF-κB pathway in *H. pylori*-associated gastric tumorigenesis. The activity of NF-κB was shown to be markedly increased in the lamina propria and epithelium of the antral mucosa of *H. pylori*-infected adults compared to those of uninfected controls, suggesting that neutrophil infiltration in the gastric mucosa of *H. pylori*-infected gastritis patients is attributed to activation of the NF-κB pathway (89). Twenty years ago, it was discovered that *H. pylori* infection increased NF-κB activity and the nuclear translocation of NF-κB p50/p65 heterodimers and p50 homodimers in transformed gastric epithelial AGS cells. Furthermore, activation of the NF-κB pathway by *H. pylori* infection could lead to the induction of proinflammatory cytokines such as IL-8 and IL-17 (90, 91). Consistently, the importance of NF-κB in *H. pylori* infection-induced gastric neoplasia was confirmed in animal models (92).

Upon sensing pathogenic *H. pylori*, cellular pattern recognition receptors (PRRs) induce intracellular signaling pathways in the innate immune response. TLRs and NOD1 are common PRRs involved in activation of the NF-κB signaling pathway (93). It was reported that infection with *H. pylori* strain 26695 increased NF-κB activity and chemokine gene expression in HEK293 and gastric epithelial MKN45 cells transfected with TLR2 and TLR5 but not TLR4 (94). NOD1 specifically recognizes peptidoglycan (PGN) delivered by the *H. pylori* cag pathogenicity island. Experimental studies, both *in vivo* and *in vitro*, have indicated that NOD1 induces NF-κB activity in response to pathogenic *H. pylori*, which is implicated in gastric inflammation and malignant lesions (95, 96). The virulence factor CagA has been identified to be responsible for the NF-κB-induced response subsequent to NOD1 activation (97).

Gastric chronic inflammation caused by the NF-κB pathway in response to *H. pylori* infection finally contributes to gastric carcinogenesis through accumulated DNA damage and abnormal cell polarity and proliferation. *H. pylori* infection induces DNA damage to exert genotoxic effects (98). Hartung et al. reported that the induction of DNA double-strand breaks (DSBs) is associated with activation of the NF-κB signaling pathway. DSBs were greatly reduced in AGS cells treated with p65/RelA RNAi or an NF-κB inhibitor. Similarly, the loss of β1 integrin resulted in decreased DSBs and inhibited IL-8 secretion after infection with *H. pylori*. Intriguingly, *H. pylori*-induced DNA damage can promote NF-κB target gene transactivation and host cell survival (99).

Moreover, NF-κB drives cell proliferation, which promotes metaplastic hyperplasia and neoplasia in the stomach. *H. pylori* infection has been shown to induce the activity of NF-κB and AP-1, which in turn promotes the expression of oncogenes (β-catenin, c-myc) and mediates the hyperproliferation of gastric cells (100). DARPP-32 has been identified as a transcriptional target gene of the NF-κB pathway that is significantly upregulated following *H. pylori* infection. Consequently, induction of DARPP-32 counteracted *H. pylori*-induced cell death and promoted gastric cell survival through the activation of AKT (101). Small RNAs such as miRNA-223-3p have been documented to link NF-κB, cellular proliferation and gastric carcinogenesis (102). As a result, *H. pylori* infection rapidly leads to activation of the NF-κB pathway, which triggers various molecular mechanisms to affect gastric neoplastic lesions.

In addition to *H. pylori*, several other microorganisms exhibit distinct functions in the GI tract. Many probiotics have been shown to inhibit the development of gastric diseases. Hwang et al. reported that *Lactobacillus casei* (*L. casei*) extract suppressed the NF-κB pathway by decreasing the expression of NF-κB p65 and IκB, which in turn induced apoptosis and inhibited the growth of gastric cancer cells (103). Infection with the non-*pylori Helicobacter* species *H. felis* links the IKKβ/NF-κB pathway to gastric inflammation and tumorigenesis. Shibata et al. generated mice in which IKKβ was conditionally deleted in gastric epithelial cells and myeloid cells to determine the role of IKKβ/NF-κB signaling in *H. felis* infection-associated gastric neoplasia. They found that deletion of IKKβ in gastric epithelial cells resulted in increased apoptosis, the accumulation of ROS and DNA damage, severe inflammation and more rapid progression to gastric preneoplasia (104).

## Modulation of the NF-κB Signaling Pathway by the Intestinal Microbiota in CRC

### *F. nucleatum* and the NF-κB Signaling Pathway

Multiple studies over the past few years have definitively addressed the carcinogenic properties of *F. nucleatum* in the initiation and development of CRC. It has become clear that *F. nucleatum* infection modulates the NF-κB signaling pathway, targeting downstream genes that regulate various cellular processes, such as the inflammatory response, cell proliferation, and cell migration, and finally affecting tumorigenesis (105–107). In the APC^Min/+^ mouse model, *F. nucleatum* treatment accelerated small intestinal and colonic tumorigenesis. Increased nuclear translocation of the p65 NF-κB subunit was observed in tumors (106). *F. nucleatum* infection can rapidly induce the host innate immune response, which activates the NF-κB signaling pathway. Several studies have shown that the common TLR4/MYD88 innate immune signaling pathway is activated following *F. nucleatum* infection in CRC cells (75). Yang et al. found that infection with *F. nucleatum* significantly activated the TLR4/MyD88 pathway to upregulate the activity of NF-κB p65 and p50 in HCT116 CRC cells. Then, miR-21, an oncogenic target miRNA in cancer, was found to be increased by *F. nucleatum* through binding to NF-κB, which thereby promoted cell survival and invasion. Inhibition of NF-κB impaired *F. nucleatum*-induced CRC cell proliferation and cell invasion. Hyperactivation of NF-κB was found in CRC tissues with high amounts of *F. nucleatum* (107). NLRX1 is a member of the NLR family that plays an important role in host innate immunity. NLRX1 was shown to negatively modulate inflammatory cytokine IL-8 expression via the transcriptional factor NF-κB in response to *F. nucleatum* infection (108). Additionally, induction of NF-κB by *F. nucleatum* infection facilitated ROS generation and production of the proinflammatory cytokines TNF-α and IL-1β (109).

Ulcerative colitis (UC) is a major type of IBD and a known risk factor for CRC (108). *F. nucleatum* was more highly enriched in human UC tissues than in normal tissues. Mechanistically, experimental studies from cells and animal models have shown that *F. nucleatum* infection activates the canonical NF-κB pathway through increasing phosphorylation levels of the NF-κB subunits p65 and IκB-α. The intestinal epithelial barrier marker ZO-1, a downstream target gene of the NF-κB pathway, was significantly decreased following *F. nucleatum* infection. Furthermore, pretreatment with human anti-IL-17F antibody attenuated *F. nucleatum*-induced NF-κB activity and intestinal inflammation, which suggests that *F. nucleatum* activates the NF-κB pathway via IL-17F (110). In addition, *F. nucleatum* was found to be more abundant in the patients with Crohn's diseases, which is another common type of IBD*. F. nucleatum* infection could promote intestinal mucosal barrier destruction during the development of Crohn's diseases (111). *F. nucleatum* is a heterogeneous species with five proposed subspecies *(ssp.)*, i.e., *ssp. animalis, ssp. fusiforme, ssp. nucleatum, ssp. polymorphum*, and *ssp. vincentii*, which show the pathogenic differences. Among the five subspecies, *ssp. fusiforme* and *ssp. vincentii* are more frequently associated with health while *ssp. nucleatum* associated with diseases (112, 113). Adherence and invasion are essential mechanisms for antimicrobial host defense mechanism and induction of inflammatory response. The invasiveness of *F. nucleatum* varies widely among different strains, and has been shown as directly related to IBD disease status (114).

### *E. coli* and the NF-κB Signaling Pathway

*E. coli* is one of the most common bacterial species that colonizes the human GI tract. Numerous studies have identified *E. coli* as a risk factor for the development of Crohn's disease, ulcerative colitis, and CRC. Enteropathogenic and enterohemorrhagic *E. coli* use a type III secretion system (T3SS) to transport dozens of effector proteins into host cells; these effector proteins in turn manipulate the host inflammatory response through activation of the NF-κB pathway (115). This bacterial pathogen has developed various mechanisms to regulate the activation of NF-κB. Pallett et al. found that the highly conserved non-LEE (locus of enterocyte effacement)-encoded effector (NleF) is responsible for nuclear translocation of the NF-κB p65 subunit and IL-8 production (116). Sahu et al. reported that non-pathogenic *E. coli* has emerged as a tumor promoter that enhances oncogenicity through enrichment of the cancer stem cell population. Mechanistically, internalization of *E. coli* leads to the activation of NF-κB through increased phosphorylation of the NF-κB subunit RelA/p65 and IKKα, inactivation of IκBα, and induction of the Wnt/β-catenin pathway through the upregulation of β-catenin and its downstream genes. Then, NF-κB and Wnt/β-catenin synergistically promote tumorigenic stemness traits (117). In addition, high NF-κB expression has been demonstrated in *E. coli*-associated IBD patients. *E. coli* strains isolated from IBD patients were found to induce the NF-κB and TNF-α promoters in HT-29 human colonic cells (118). Karrasch et al. determined the role of the TLR/NF-κB signaling pathway in bacteria-induced colitis using animal models—IL-10-deficient mice and NF-κB knock-in mice. As a result, coinfection with the commensal bacterial strain *Enterococcus faecalis* led to experimental colitis through activation of the TLR/NF-κB signaling pathway (119). Taken together, these results show that some *E. coli* strains, in which the NF-κB pathway induces chronic inflammation, play a crucial role in colorectal carcinogenesis.

### *P. anaerobius* and the NF-κB Signaling Pathway

*P. anaerobius* is a gram-positive anaerobic bacterium that was recently identified to be especially enriched in the stool samples of CRC patients. Jun Yu et al. from the Chinese University of Hong Kong recently undertook research to determine the role of *P. anaerobius* and its molecular mechanism in colorectal carcinogenesis. *P. anaerobius* was found to induce the production of intracellular ROS through its interaction with TLR2 and TLR4. As a result, total cholesterol synthesis and the proliferation of intestinal epithelial cells were significantly enhanced (81). Similar to other bacteria, *P. anaerobius* adheres to the intestinal mucosa and encodes a virulence factor to induce the host immune response. Putative cell wall binding repeat 2 (PCWBR2), a *P. anaerobius* surface protein, interacts with the corresponding cell surface receptor integrin α2/β1. Subsequently, experimental data from CRC cells and *APC*^*Min*/+^ mice indicated that *P. anaerobius* challenge activated the PI3K/AKT and NF-κB signaling pathways via phosphorylation of the AKT and p65 NF-κB subunits. As a result, *P. anaerobius* modulates the tumor immune microenvironment, including the expansion of myeloid-derived suppressor cells, tumor-associated macrophages and granulocytic tumor-associated neutrophils. Cell proliferation and the proinflammatory response were significantly increased by *P. anaerobius* infection, further accelerating colorectal tumorigenesis (120).

### Enterotoxigenic *B. fragilis* and the NF-κB Signaling Pathway

Enterotoxigenic *B. fragilis*, but not non-toxigenic *B. fragilis*, is associated with the development of intestinal diseases, such as human inflammatory diarrhea and colorectal carcinogenesis. Enterotoxigenic *B. fragilis* targets intestinal epithelial cells by producing *BFT*. As a result, cells develop molecular mechanisms to activate the NF-κB signaling pathway. To explore the role of BFT in enterotoxigenic *B. fragilis*-triggered tumorigenesis. Chung et al. constructed Apc^Min^ mice colonized with an enterotoxigenic B. fragilis strain possessing an in-frame chromosomal deletion of *bft* gene. The results showed that *B. fragilis* stimulated intracellular IL-17 secretion to activate the NF-κB pathway, which in turn induced the expression of chemokines (CXCL1, CXCL2, and CXCL5) that collectively contributed to colonic carcinogenesis (121, 122). The cytokine IL-8, a key downstream target gene of NF-κB, was also significantly increased in intestinal epithelial cells treated with active enterotoxigenic *B. fragilis* (123). The β-catenin and GSK3β cellular signaling pathways are involved in NF-κB activity and IL-8 expression in *B. fragilis*-infected cells (124). Host tissues recruit inflammatory cells to induce sustained inflammation through activation of the NF-κB signaling pathway in pathological processes and increase the risk of CRC through aberrant regulation of other cellular processes, such as cell proliferation and angiogenesis.

## Concluding Remarks

The gastrointestinal microbiota plays an important role in maintaining host physiological processes. Aberration of the microbiota might ultimately result in various diseases such as metabolic, cardiovascular, immune, mental, and gastrointestinal diseases or even cancer. The causal relationship between gastrointestinal cancer and the microbiota has been gradually validated. Indeed, multiple studies suggest that utilizing the microbiota, especially specific bacteria, may provide novel microbial biomarkers for prevention, diagnosis and treatment. Currently, some clinical trials based on these microbes (e.g., *F. nucleatum*)are ongoing. Moreover, the activated NF-κB signaling pathway is considered an important line of defense against microbial pathogens. Abnormal and sustained activation of NF-κB signaling contributes to malignant transformation from inflammation to cancer. Therefore, selectively targeting molecules of the NF-κB signaling pathway to block the association between pathogens and NF-κB shows therapeutic potential and benefit in cancer treatment. For example, specific NF-κB inhibitors targeting the IKK complex have shown promise as anticancer therapeutics (12). However, beyond the involvement of NF-κB, the relationship between the microbiota and gastrointestinal carcinogenesis is very complex. Moving forward, there is a need to explore and more deeply understand the underlying mechanisms that link the microbiota and host response.

In addition, molecular pathological epidemiology (MPE) that investigate the interactive effect of some specific molecular features and environmental factors on disease prognosis and clinical outcome has been widely applied to cancer research (125, 126). The host genetic, diet, lifestyle and other environmental factors, which have been closely linked with gut microbiota, are critical for the prevention of gastrointestinal cancer. Modifications of the western lifestyles such as high fat diet that resulting in overweight or obesity could substantially reduce the CRC incidence (127). Eating less salted or pickled foods is considered to be important for prevention of GC (128). This promising direction may help to gain new insights into the pathogenic process, prevention and treatment of gastrointestinal cancer.

## Author Contributions

NLi, CP, and NLu discussed the contents. NLi, CP, and YO wrote and edited this manuscript. NLi supervised and oversaw the manuscript. All authors read and approved the final manuscript.

## Conflict of Interest

The authors declare that the research was conducted in the absence of any commercial or financial relationships that could be construed as a potential conflict of interest.
